# Factors related to human-vector contact that modify the likelihood of malaria transmission during a contained *Plasmodium falciparum* outbreak in Praia, Cabo Verde

**DOI:** 10.3389/fepid.2022.1031230

**Published:** 2022-11-25

**Authors:** Gillian Stresman, Adilson José DePina, Luca Nelli, Davidson D. S. Monteiro, Silvânia da Veiga Leal, António Lima Moreira, Ullardina Domingos Furtado, Jerlie C. Loko Roka, John Neatherlin, Carolina Gomes, Abderrahmane Kharchi Tfeil, Kimberly A. Lindblade

**Affiliations:** ^1^Department of Infection Biology, London School of Hygiene and Tropical Medicine, London, United Kingdom; ^2^College of Public Health, University of South Florida, Tampa, FL, United States; ^3^National Malaria Elimination Program, CCS-SIDA, Praia, Cabo Verde; ^4^School of Biodiversity, One Health, and Veterinary Medicine, University of Glasgow, Glasgow, United Kingdom; ^5^Laboratório de Entomologia Médica, Instituto Nacional de Saúde Pública, Praia, Cabo Verde; ^6^Praia Health Delegation, Ministry of Health, Praia, Cabo Verde; ^7^U.S. Centers for Disease Control and Prevention, Dakar, Senegal; ^8^U.S. Centers for Disease Control and Prevention, Atlanta, GA, United States; ^9^World Health Organization, Praia, Cabo Verde; ^10^World Health Organization Intercountry Support Team, Ouagadougou, Burkina Faso; ^11^World Health Organization, Geneva, Switzerland

**Keywords:** malaria, transmission reduction, transmission heterogeneity, reproductive number, Cabo Verde

## Abstract

**Background:**

Determining the reproductive rate and how it varies over time and space (R_T_) provides important insight to understand transmission of a given disease and inform optimal strategies for controlling or eliminating it. Estimating R_T_ for malaria is difficult partly due to the widespread use of interventions and immunity to disease masking incident infections. A malaria outbreak in Praia, Cabo Verde in 2017 provided a unique opportunity to estimate R_T_ directly, providing a proxy for the intensity of vector-human contact and measure the impact of vector control measures.

**Methods:**

Out of 442 confirmed malaria cases reported in 2017 in Praia, 321 (73%) were geolocated and informed this analysis. R_T_ was calculated using the joint likelihood of transmission between two cases, based on the time (serial interval) and physical distance (spatial interval) between them. Log-linear regression was used to estimate factors associated with changes in R_T_, including the impact of vector control interventions. A geostatistical model was developed to highlight areas receptive to transmission where vector control activities could be focused in future to prevent or interrupt transmission.

**Results:**

The R_T_ from individual cases ranged between 0 and 11 with a median serial- and spatial-interval of 34 days [interquartile range (IQR): 17–52] and 1,347 m (IQR: 832–1,985 m), respectively. The number of households receiving indoor residual spraying (IRS) 4 weeks prior was associated with a reduction in R_T_ by 0.84 [95% confidence interval (CI) 0.80–0.89; *p*-value <0.001] in the peak-and post-epidemic compared to the pre-epidemic period.

**Conclusions:**

Identifying the effect of reduced human-vector contact through IRS is essential to determining optimal intervention strategies that modify the likelihood of malaria transmission and can inform optimal intervention strategies to accelerate time to elimination. The distance within which two cases are plausibly linked is important for the potential scale of any reactive interventions as well as classifying infections as imported or introduced and confirming malaria elimination.

## Introduction

Estimating the average number of secondary infections per infected individual (R_0_) in a population provides an important measure of transmission intensity and provides insight into the frequency with which humans come into contact with infectious vectors (1). R_0_ is known to vary over time and space according to the natural history and ecology of a given infection and the implementation of effective control interventions. Once interventions are in place, the transmission potential of an infection changes and rather than R_0_, the appropriate term becomes the effective reproductive number (R_T_) (2). Therefore, identifying where transmission potential is high (R_T_ > 1) and where it is low (R_T_ < 1) provides important insight to better understand what factors drive transmission and improve targeting of additional control or elimination strategies (3).

Malaria is a vector-borne disease caused by the *Plasmodium* parasite and transmitted by *Anopheles* mosquito vectors. *Plasmodium falciparum* is the most common cause of malaria across the African continent and is associated with significant morbidity and mortality. Research suggests that areas with a higher density of the mosquito vector are associated with a higher risk of malaria (4). This finding is not surprising given that areas with a greater likelihood of contact between humans and mosquito vectors will result in higher rates of malaria transmission. However, the research to date on understanding malaria transmission dynamics relies on use of mathematical models that are not always based on empirical data (5). Where primary data exist to assess malaria transmission dynamics, it is confounded by the use of vector control interventions in the population that modifies transmission (6), reliance on entomological data that does not directly measure host-seeking vectors (7), or presence of malaria immunity masking incident infections making it difficult to measure a transmission event (8–10). A better understanding of the spatial and temporal transmissibility of *P. falciparum* in the absence of control or significant anti-malarial immunity will help assess malaria transmission potential and inform appropriate control and elimination strategies.

Malaria transmission in Cabo Verde, an archipelago off the coast of West Africa, has been very low for decades (11). Elimination of malaria has occurred twice in Cabo Verde, but each time, transmission was re-established in subsequent years. Clinical incidence in Praia, the capital city that has historically had the highest incidence of malaria, has been <1 per 1,000 population at risk since 2010 and prior to 2017, the last reported epidemic was in 1987 (12). Therefore, the population of Praia has negligible levels of protective immunity to malaria and the majority of residents would be expected to develop clinical symptoms upon infection (13, 14). By capturing all clinical infections, a useful proxy for a contact between a human and an infectious vector, the outbreak that occurred between July 2017 and January 2018 provides a unique opportunity to directly estimate R_T_ for malaria in a sub-Saharan African setting with typical African malaria vectors. Therefore, the aims of this work were to estimate R_T_ for malaria in Praia, Cabo Verde to characterize the spatiotemporal variation in R_T_, identifying areas where malaria transmission potential is high (i.e., the predicted R_T_ was >1), and determine factors associated with a change in R_T_ as the epidemic progressed.

## Methods

### Overview of the 2017 outbreak

In 2017, there were 442 *P. falciparum* malaria cases identified in Praia, of which 97.7% were reported between July and December. The epidemiology and risk factors associated with malaria cases have been described (15). In addition to the routine prompt, supervised treatment of all cases with artemether-lumefantrine and a single low-dose of primaquine as well as larviciding to contain the outbreak, the malaria programme responded with two main interventions: indoor residual spraying (IRS) with deltamethrin (K-othrine WP50, Bayer, South Africa) of houses in the neighborhoods where cases were identified, and reactive case detection (RACD), that is, testing those individuals residing with or near confirmed cases for malaria by rapid diagnostic test (RDT) and microscopy and treating those testing positive. Of note, the RACD activity identified seven individuals positive for malaria, all of whom were symptomatic within a day of being tested as part of the RACD response. For all cases, the median number of days between symptom onset and seeking care was 2 days (IQR: 0–3 days) and the majority of cases were in adults (median age 30 years, IQR: 20–43 years) and males (65.7%).

### Epidemiological data

The data routinely collected on each malaria case to support this analysis was provided by the National Malaria Elimination Program (NMEP) in Cabo Verde. Individuals seeking care at health centers or private clinics and suspected to have malaria, or who tested positive by RDT, were referred to the principal hospital in Praia for malaria microscopy and treatment. Most cases were hospitalized for 3 days to supervise treatment. Cases were investigated by Health Delegation personnel either in the hospital or at their residence. Key variables collected included demographic details of each case (e.g., age, sex), neighborhood of residence, date of symptom onset, date of being tested for malaria, and travel history. In Cabo Verde, cases are classified as imported if the individual reports travel to a malaria endemic country within the previous 4 weeks, and as locally-acquired if otherwise (12). The available data consisted of 10 cases reported between January and June 2017 and 432 cases from July through December 2017. The final case reported during the epidemic occurred on January 8th, 2018 but the data were not available for this analysis. No entomological data on adult mosquitoes was collected as part of the programmatic response and thus was not available to include in this analysis. After the epidemic was contained, a retrospective mapping activity was undertaken with the objective of geolocating the residences of all confirmed malaria cases reported in 2017. The information collected by the surveillance system, i.e., addresses and phone numbers, were used to trace the cases; 321 (72.6%) of the residences of the malaria cases were successfully located, mapped and made available for analysis. In addition to the routine malaria surveillance data, the NMEP provided data on the malaria control interventions implemented in Praia in 2017. The data consisted of the number of structures sprayed with IRS and the number of people tested as part of RACD per week.

### Data analysis

Individual-level estimates of R_T_ were obtained using the methods developed by Routledge et al. (16). Briefly, the model estimates the joint likelihood of transmission between two malaria cases according to the most probable network structure making up the underlying transmission chain. First, the serial interval was estimated based on the time between a case showing symptoms and the subsequent case they may infect showing symptoms. This is estimated according to the normalized likelihood of a shifted Rayleigh distribution with uninformed priors. Next, the likelihood of transmission is estimated by maximizing the conditional of the underlying transmission chain based on the estimated serial interval and distance between the two cases (Supplementary Figure 1). The underlying transmission network is governed by the following assumptions: (1) locally-acquired cases can infect others and be infected whereas imported cases can only infect others; (2) onward transmission occurs after the case becomes symptomatic; (3) all infections identified receive curative treatment and therefore, are removed from the potential infectious reservoir and; (4) people can be infected by those undetected as part of the routine surveillance activities. The estimated R_T_ is therefore the sum of the conditional likelihoods of the cases being associated with each infection over time (17). The median and interquartile range (IQR) of the serial interval between infection pairs with R_T_ >0 was calculated (16). A similar approach was applied to estimate the “spatial serial interval,” or the median distance between two paired cases.

Exploratory analysis was conducted to assess the spatial and temporal trends in the estimated R_T_ and the corresponding herd immunity threshold was calculated. As malaria does not confer sterilizing immunity, this term is defined here as the proportion of the population that needs to be treated or covered by an intervention that fully protects users from infections to interrupt transmission. Log-linear regression was used to identify whether the number of structures sprayed with IRS or people tested as part of RACD had an impact on R_T_. To account for the time between the application of intervention and the potential impact on transmission, lag periods of 1-, 2-, 3-, and 4-weeks prior were tested for each intervention. To account for the strong temporal trend associated with the malaria epidemic, a factor variable accounting for the pre- (January–June; weeks 0–26), peak- (July–September; weeks 27–39), and post-epidemic (October–December; weeks 40–52) period, as well as a non-linear effect of week, were tested. Interactions and multiple model forms including generalized additive models were tested with the Akaike Information Criteria (AIC) determining the optimal model fit. All analysis was conducted in R statistical software (Version 4.1.1).

Geostatistical modeling was undertaken to develop a map of predicted R_T_ in Praia as a function of candidate environmental predictors, namely altitude, land use, and distance from inland surface water (rivers, streams, and ditches). Digital elevation model imagery for Praia was obtained from TerraSAR-X/TanDEM-X (18). Land use classification of high-resolution satellite imagery was conducted using a random forest supervised classification algorithm (19). Briefly, a multi-spectral (eight bands Panchromatic: 450–800 nm) pan-sharpened QuickBird-2 satellite imagery, with 30 cm resolution, was obtained for November 30th, 2017. After preliminary unsupervised classification, five land use categories were defined: built-up areas, dense vegetation, sparse vegetation, bare ground, and roads. For each category 200 training points were digitized, supervised classification employing a decision tree algorithm was performed using the *rpart* package in R (20). Classification accuracy was assessed *via* error rate estimates and confusion matrices. Land use categories were then measured as proportion within a circular buffer around each point, with different radii (25, 50, 75, and 100 m). Water courses, including ditches and canals visible on the imagery were digitized and a raster layer of distance from the nearest water course was created.

To generate a map of R_T_ in Praia, a spatially explicit zero-inflated negative binomial generalized linear mixed model (GLMM) was developed, using the package *glmmTMB* in R (21). Spatial autocorrelation was included as a spatial random effect and modeled using a multivariate normal distribution with a mean vector of zero and a covariance matrix defined by a Matérn correlation function with pairwise correlations at the scaled Euclidean distance between coordinates. Models with land use classes aggregated to four spatial scales (25, 50, 75, and 100 m), altitude and distance from water were tested and included in both the conditional model and in the zero-inflated component. The AIC determined the best fitting model to generate a map of the estimated R_T_ across Praia (Supplementary Figure 1).

This research received ethical approval from the Comité Nacional de Ética em Pesquisa para a Saúde (72/2020), the London School of Hygiene and Tropical Medicine (21503), and the World Health Organization (005473). The data used in this analysis were collected as part of the routine malaria surveillance activities and the epidemic response; therefore, informed consent was not obtained (15). All identifying information was removed from the database except for the household location before analysis. To preserve anonymity of individuals, error terms were introduced in all visualizations. Results were presented according to the RECORD checklist for studies using routinely collected health data (Supplementary File 1) (22).

## Results

**Figure 1:**
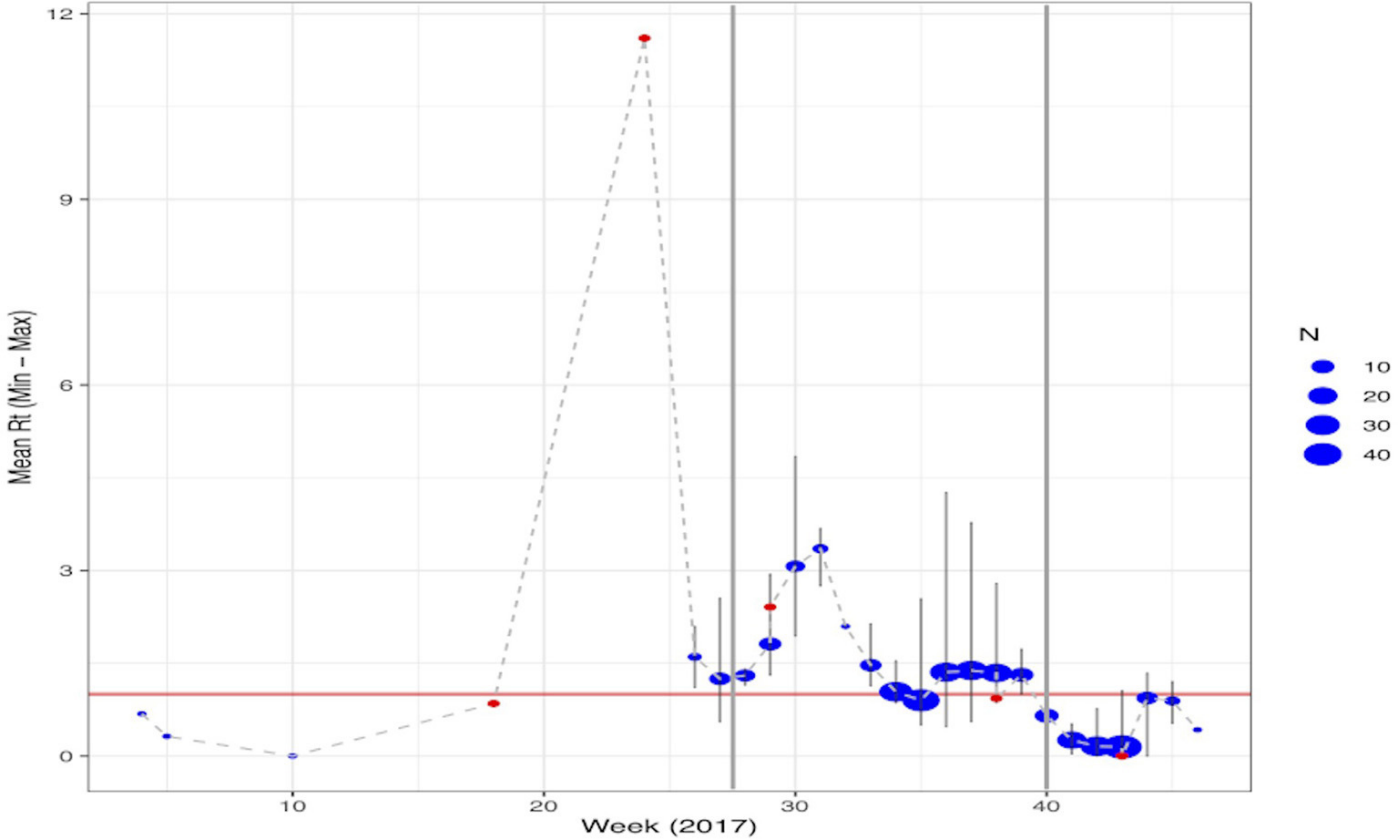
Temporal trend of the estimated mean R_T_ per week of cases reported through 2017. Malaria cases classified as imported are shown in red with locally acquired infections in blue. The size of the points represents the number of cases reported in that week with the maximum and minimum estimated R_T_ highlighted by the light gray lines. Points where the range is not shown reflect only having a single case reported that week. The vertical gray lines represent the different phases of the epidemic: Pre-epidemic from January June or weeks 0–26; Peak-epidemic from July to September or weeks 27–39 and; Post-epidemic from October December or weeks 40–52.

Of the 321 malaria cases in 2017 that were geolocated, five were imported and 315 were diagnosed between July and December. Over the course of the outbreak, the estimated R_T_ ranged from 0 to 11 (Supplementary Figure 2). For locally-acquired infections, the estimated R_T_ ranged between 0 and 4 with 33.0% of infections having R_T_ >1 whereas for imported infections the estimated R_T_ ranged between 0 and 11 with 29.1% of infections having R_T_ >1. During the pre-epidemic period, the weekly mean R_T_ was consistently below one; the peak-epidemic period was initiated by an imported case with an R_T_ of 11 and the R_T_ stayed above one until week 35 and during the post-epidemic period after interventions were scaled-up, the maximum R_T_ was 4. An R_T_ of 11 and 4, or the maximum transmission potential of infections pre- and post-IRS scale-up, corresponds with herd immunity thresholds of 90.9% [i.e., (1-(1/11)] and 75.0%, respectively. There was a second wave of R_T_>1 between week 36 and 39. The post-epidemic period started in week 40 and mean weekly R_T_ remained <1 through December 2017 (Figure 1). The median serial interval was estimated to be 34 days (IQR: 17–55 days), consistent with values previously reported (16). The estimated spatial serial interval in this area was 1,347 m (IQR: 832–1,985 m).

**Figure 2:**
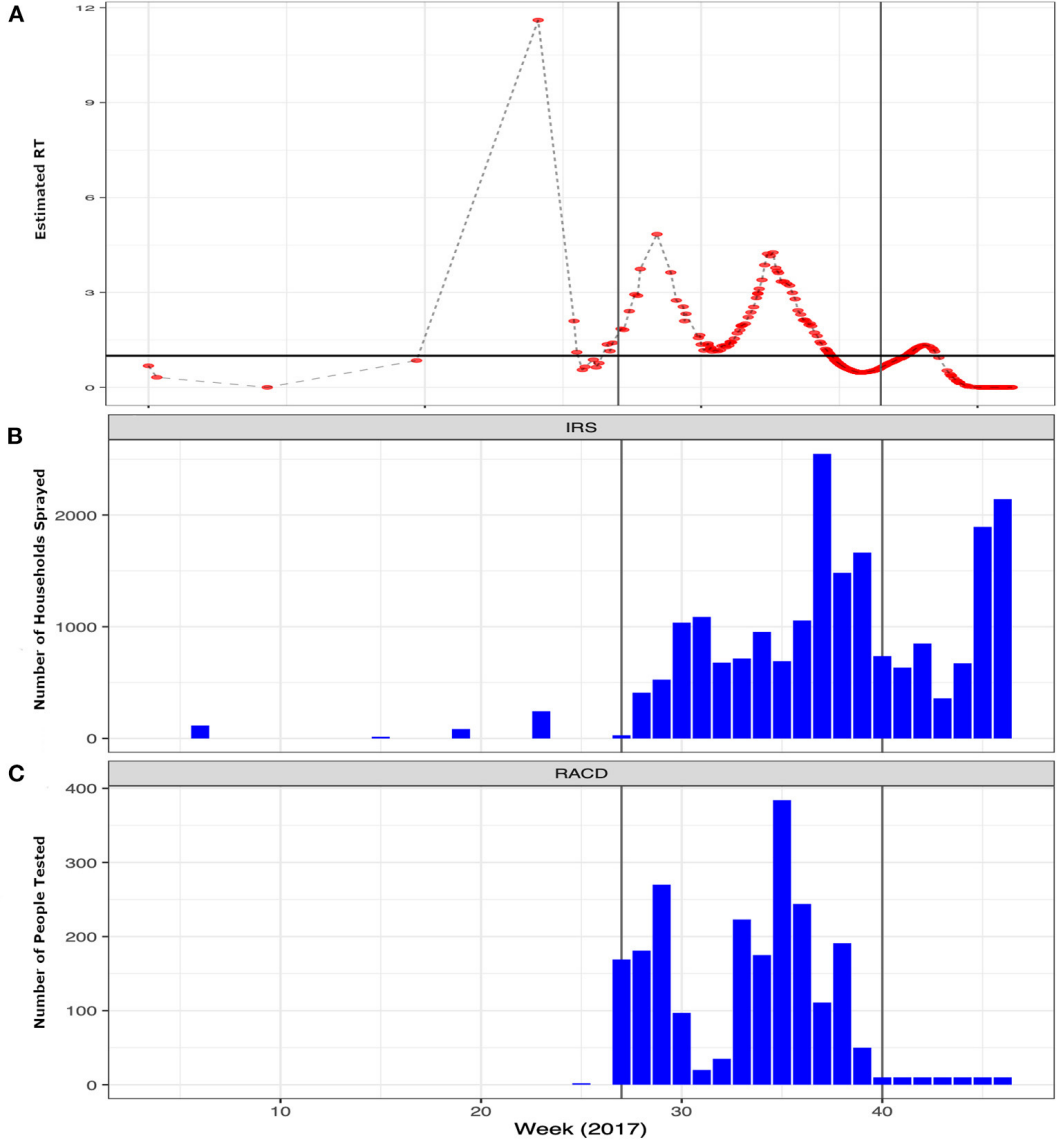
Weekly trends of the epidemic and interventions implemented by the program to contain the outbreak. **(A)** Temporal trends in the estimated Rt per case (red points) over the course of 2017 highlighting the epidemic period. The x-axis shows the weeks starting in 2017 through the end of the pandemic with the y-axis showing the estimated Rt. **(B)** The number of structures sprayed with indoor residual spray (IRS) and **(C)** the number of people tested per week as part of reactive case detection (RACD) activities per week over the same time period. The vertical gray lines represent the different phases of the epidemic: Pre-epidemic from January June or weeks 0–26; Peak-epidemic from July September or weeks 27–39 and; Post-epidemic from October December or weeks 40–52.

During the pre-epidemic period only 105 houses received IRS and no one was tested as part of a RACD investigation. In contrast, after the epidemic was confirmed in July and through the end of the post-epidemic period, 30,840 houses received IRS and 2,220 people participated in the RACD response (Figure 2). The results of the final log-linear regression model suggest that R_T_, or the number of secondary cases associated with each primary case, declined by 0.89 (95% CI: 0.87–0.93) on average, per week, and that R_T_ was significantly higher during the peak epidemic period compared to the pre-epidemic period (67.44, 95% CI: 20.38–223.20). There was a significant interaction between the number of houses that received IRS 4 weeks prior and the epidemic period; there was no association between IRS and R_T_ during the pre-epidemic period while during the periods of peak- and post-epidemic, the number of houses sprayed, accounting for a 4-week lag, was significantly associated with a reduction in R_T_ (Table 1). There were distinct spatio-temporal trends observed over the course of the epidemic (Supplementary Movie 1). The few malaria cases that were confirmed in the pre-epidemic period were scattered throughout the city. With the onset of the epidemic in July, explosive waves of cases are visible and shifting slightly north before being contained.

**Table 1:** Results of the log linear regression to assess the impact of the interventions on the estimated R_T_ over the course of the epidemic.

**Variable**	**β**	**95% confidence interval**	***P*-Value**
Intercept	0.59	0.24–1.47	0.26
Week	0.89	0.85–0.93	< 0.001
**Epidemic period**			
Pre-epidemic (January–June)	1	–	–
Peak-epidemic (July–September)	67.44	20.38–223.20	< 0.001
Post-epidemic (October–December)	4.55	0.94–22.05	0.06
IRS – 4-week lag	1.19	1.12–1.25	< 0.001
**Epidemic period: IRS 4-week lag interaction**			
Pre-epidemic:IRS	1	–	–
Peak-epidemic:IRS	0.84	0.80–0.89	< 0.001
Post-epidemic:IRS	0.84	0.80–0.89	< 0.001

β, model coefficients indicating the expected change in R_T_. The corresponding 95% confidence intervals and p-values are shown.

**Figure 3:**
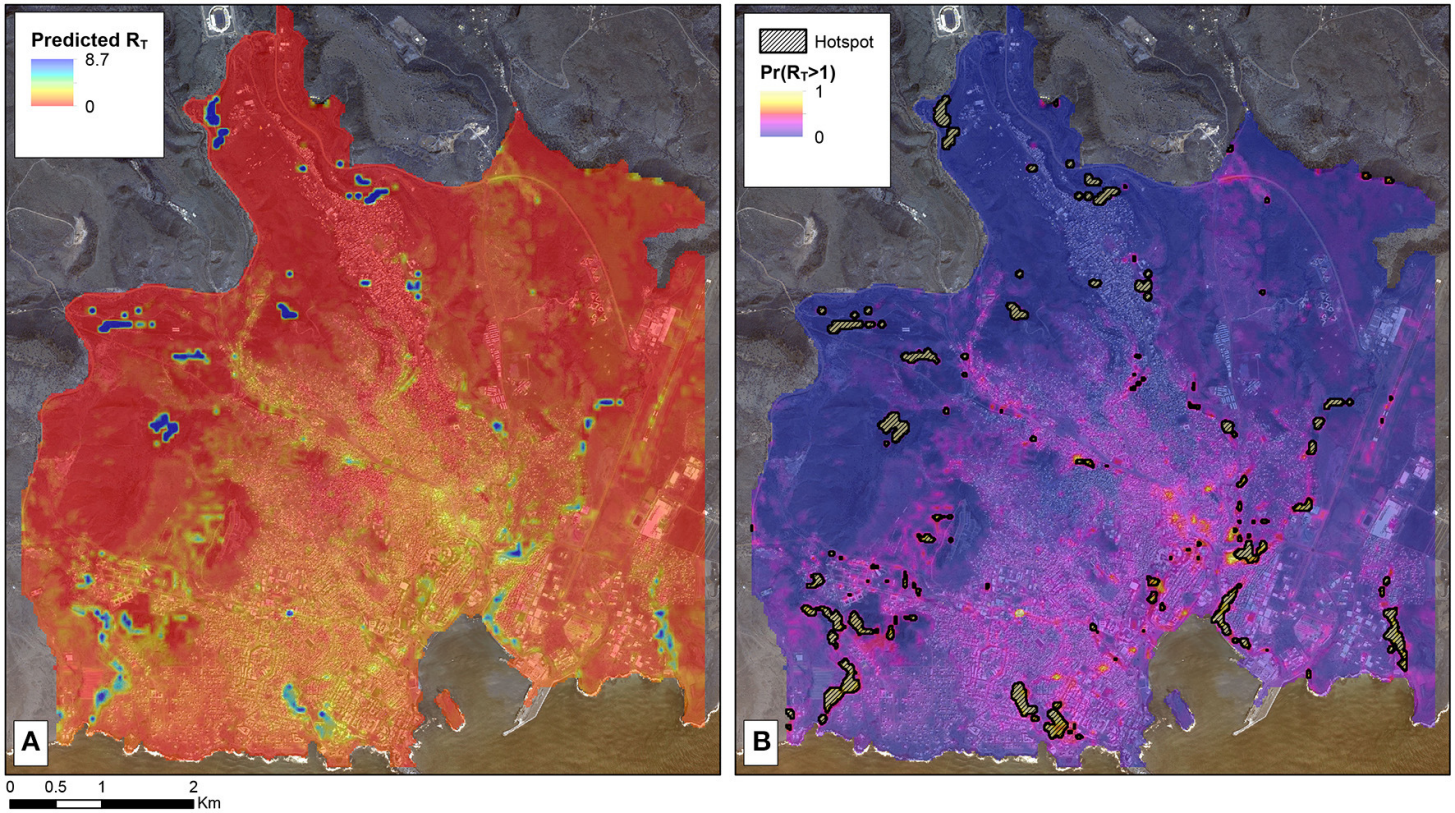
Map of malaria receptivity in Praia, Cabo Verde based on the 2017 outbreak. **(A)** Map of the estimated R_T_ as predicted by the geostatistical model informed by environmental covariates as predictors. **(B)** The probability that the area has a predicted R_T_ > 1. The areas where there is at least 80% probability that R_T_ is > 1 are marked by the black hashed areas. This arbitrary threshold was selected for visualization purposes, with the specific areas of programmatic interest being dependent on the goals of the program and resources available. These areas are thus ones where malaria is particularly receptive and may spark future epidemics if parasites are imported when the *Anopheles* mosquito vectors are active.

The 25 m spatial scale was the optimal scale for the land-use classification algorithm (Supplementary Table 1). The geostatistical model that best fit the data (Supplementary Figures 3–5) suggested that areas with a higher proportion of bare ground were associated with lower RT than areas with dense or sparse vegetation. In addition, proximity to surface water, such as canals, and a lower altitude were associated with an increase in the expected R_T_ (Table 2; Supplementary Figure 6). The map of the predicted R_T_ in Praia (**A**) and the predicted probability that R_T_ was >1 (**B**) are shown in Figure 3.

**Table 2:** Estimates for the fixed effects of best model explaining R_T_ values in Praia (zero-inflated negative binomial model, with spatial random effect).

**Variable**	**β**	**SE**	**LCI**	**UCI**	***P*-Value**
Intercept	−1.02	0.17	−1.35	−0.69	< 0.001
Bare ground	−0.40	0.19	−0.77	−0.04	0.031
Buildings	−0.06	0.22	−0.50	0.38	0.775
Dense vegetation	0.06	0.18	−0.30	0.41	0.753
Sparse vegetation	0.21	0.14	−0.06	0.49	0.122
Distance from water	−0.23	0.14	−0.51	0.04	0.098
Altitude	0.32	0.20	−0.08	0.71	0.114
**Zero inflation**
Intercept	−6.36	1.96	−10.19	−2.53	0.001
Bare ground	−1.45	1.19	−3.77	0.88	0.223
Buildings	1.96	1.38	−0.75	4.67	0.157
Dense vegetation	−2.53	2.37	−7.17	2.11	0.284
Sparse vegetation	1.83	1.27	−0.66	4.33	0.149
Distance from water	−3.08	1.52	−6.05	−0.11	0.042
Altitude	4.61	1.51	1.65	7.57	0.002

β, model coefficients; SE, standard errors; LC, 95% lower confidence intervals; UCI, 95% upper confidence intervals. Land use variables are proportions in circle of 25 m radius around each location; distance from surface water and altitude are in meters.

## Discussion

We estimated the individual-level R_T_ of malaria throughout the course of an outbreak in 2017 in Praia, Cabo Verde, a population without significant immunity to malaria and where only minimal control efforts were in place before the start of the epidemic. The epidemic consisted of three distinct waves of transmission in this urban environment with cases associated with between 0 and 11 additional malaria infections. The maximum estimated R_T_ of 11 suggests that in a non-immune population with low coverage of control interventions, the herd immunity threshold is 90.9%, but in the presence of effective vector control, here IRS, the maximum R_T_ was 4, the threshold reduced to 75.0%. This suggests that the majority of the population needs to be covered with an intervention that fully protects users from acquiring incident infections, for example uses LLINs or receives prompt treatment of any incident infection before onward transmission is possible, to interrupt transmission.

In this setting, all infected individuals exhibited symptoms and care was sought within between 1 to 3 days where they received effective antimalarial treatment as an inpatient. This suggests that the transmission window where contact with a *Anopheles* mosquito vector was very short and likely further contributed to reducing the transmission potential of the outbreak. Similarly, here all treatment was observed as part of inpatient care and ensured that all infections were cleared and likely further reduced the risk of onward transmission. In contrast, where population immunity is high or rates of care-seeking are lower or more delayed than what was observed here, the transmission window is likely larger if infections persist for longer before treatment. In this case, it is possible that R_T_ may be higher as persistent infections provide more opportunity for contact with a competent vector.

In this setting, we estimated that it can take a median of 1 month (34 days) for secondary malaria infections to become symptomatic and detected by routine surveillance. To our knowledge, this is the first time the period between when a person presents for care and the subsequent person infected in the chain becomes symptomatic. The temporal relationship observed is consistent with the expected intrinsic and extrinsic incubation period of malaria and has important programmatic implications for confirming the classification of a case as indigenous or introduced (23, 24).

We extended the construct of the serial interval (describing the time difference between two linked, symptomatic cases detected by the health system) to the concept of a serial spatial interval that describes the physical distance between the residences of two linked, symptomatic cases. To our knowledge, this is the first time that this concept has been considered or calculated for malaria. Although no data on adult mosquito densities was available to confirm this, the expected distance between linked cases is likely a combination of both human and mosquito movement and of the opportunities for intersection that allows for human-vector contact. The spatial serial interval is likely to vary depending on parasite and mosquito species, weather, population density, presence of domestic animals, housing characteristics and other factors that mediate human-vector contact. Although there will be variance in the spatial serial interval in different areas, understanding plausible values can help inform interventions conducted in response to a confirmed malaria case in areas of very low transmission as well as the classification of cases as introduced or indigenous. We found that the median spatial serial interval was 1.3 km, which is consistent with the maximum dispersal of anophelines that are not likely to be wind-aided (25). However, given the densely population urban setting in which these cases occurred, we expected to find linked cases occurring nearer to each other (26). One possible reason for finding a larger distance between linked cases than expected is that cases, which were largely adult men, could have been infected in locations other than their residences. Combined, the serial- and spatial-interval observed suggest that the interaction between human movement, mosquito movement, and the parasites' development period must be accounted for when characterizing malaria transmission and targeting a response.

Both vector control interventions and ecological variables known to be conducive to mosquito breeding were associated with changes in R_T_. Firstly, the number of households sprayed by IRS within the targeted neighborhoods 4 weeks prior, was associated with a reduction in R_T_ during the peak- and post-epidemic periods. It is possible that this association was a result of the direct impact of IRS, but as *Anopheles Arabiensis* is an exophilic vector, it could have been a proxy for unmeasured factors including changing behaviors as the epidemic progressed. Interestingly, RACD was not associated with any reduction in transmission. In a non-immune population, most, if not all, infections are expected to become symptomatic. Thus, the time between an infection being detectable using routine diagnostic tools and onset of symptoms would likely be short, leaving few infections in the community for RACD to identify. In this population, symptomatic infections were promptly detected and treated within the routine health system, as has previously been reported (27); therefore the lack of association between RACD and transmission reduction is not surprising. In other settings where residual protective immunity exists and incident infections are more difficult to detect, RACD may be associated with a change in R_T_ and thus be a more effective intervention (28).

Secondly, the geospatial analysis identified several ecological factors that improved the model fit when analyzing changes in R_T_ and delineated areas that are conducive to malaria transmission (i.e., R_T_ > 1). According to the best model fit, the variables related with increasing R_T_ included lower altitude, being closer to water bodies, and areas with vegetation whereas areas with bare ground or buildings was associated with reductions in transmission potential. Although entomological data were not available for this analysis and the sample size of the number of cases with an R_T_ value was small, the factors that were found to influence R_T_ are consistent with those that impact the capacity for mosquito development and receptivity to transmission (29). The corresponding maps identified areas where onward transmission is likely (e.g. R_T_ >1) informing where vector control interventions can be targeted in future to suppress receptivity (30). Maintaining a vigilant health system able to promptly detect and treat any and all infections is crucial to interrupting transmission. However, infections are only likely to cause onward transmission in areas receptive to malaria transmission where there is sufficient contact between infective mosquitoes and human hosts: the high estimated R_T_ observed in a single case was likely a result of this case residing in an area that was highly receptive to malaria at the right time when the mosquito vectors were abundant. This case may have been responsible for triggering the outbreak. In future, maintaining targeted vector control in areas where transmission is most likely can reduce receptivity and R_T_ (31).

There are some important limitations to this study. First, other methods for estimating *P. falciparum* R_T_ are available and could have been applied to this case study in Praia (5, 32). Although a direct comparison of models is beyond the scope of this paper, the results presented in this report are consistent with those found by other studies. For example, the distribution of the estimated R_T_ highlighted the significant heterogeneity expected in malaria transmission with a small proportion of individuals driving the majority of transmission (33). Therefore, we would expect the results of applying different methods for estimating R_T_ to yield similar findings, but without parasite genomic data, confirming the transmission chains to determine how many secondary infections resulted from each infected individual is challenging (34). Next, the data on interventions administered to contain the outbreak was collected from the NMEP. Ideally, the number of households receiving IRS or people tested as part of RACD would be available as numbers per week per neighborhood to account for both the spatial and temporal heterogeneity of the outbreak and the response. However, data were only available at a weekly resolution and, therefore, the analysis of the impact of interventions on R_T_ is ecological in nature. Despite that limitation, the results of the model are consistent with the understanding of the impact of the applied interventions on transmission suggesting the findings are plausible. The model framework assumes that imported and locally-acquired infections contribute differently to the underlying transmission chain with imported infections only contributing to onward transmission. In most programmatic settings, including in Cabo Verde, imported infections are classified as those who have traveled to a malaria endemic area in the previous 4 weeks. It is possible that the clinicians misclassified cases on whether they were imported or not which could impact the model results. However, given that clinicians were blinded to the associated R_T_ of that individual, any misclassification bias is expected to be non-differential. Next, here we are considering the herd immunity threshold is based on the maximum number of secondary infections associated with a single case. Malaria transmission is known to be heterogeneous and thus, a range of values, as was observed here, is not unexpected. However, if a single infection is capable of seeding up to 11 additional infections, coverage at anything less than this threshold would be unlikely to lead to sustainable reductions and therefore, is more meaningful than other summary measures such as the median or range. Finally, the spatial coordinates used to estimate R_T_ were of the house where the case resided and is the assumed location of the transmission event for locally-acquired infections. The *Anopheles* mosquitoes' peak biting times are between dusk and dawn making this assumption plausible; however, given that the majority of infections were in adult men as has previously been reported (15), we acknowledge that transmission could have occurred elsewhere, which may have introduced measurement bias when estimating R_T_ and the corresponding spatial serial interval. Future analysis to assess any differences in the distance between two linked cases and demographic risk factors could help refine this.

Ultimately, measuring R_T_ is difficult for malaria and transmission potential is impacted by factors affecting both the human host and mosquito vector. Understanding factors that impact transmission potential and the expected patterns whereby infectious humans come into contact with the *Anopheles* mosquito vectors will enable more precise targeting of interventions to interrupt transmission as well as improving estimates of malaria transmission potential.

## Data availability statement

The data analyzed in this study is subject to the following licenses/restrictions. The data underlying this article were provided with permission from the National Malaria Elimination Program of Cabo Verde. The anonymized, aggregated data collected to support this pooled data analysis and corresponding R code are available from the corresponding author upon reasonable request. Requests to access these datasets should be directed at: GS, gstresman@usf.edu.

## Ethics statement

This research received ethical approval from the Comité Nacional de Ética em Pesquisa para a Saúde (72/2020), the London School of Hygiene and Tropical Medicine (21503), and the World Health Organization (005473). Written informed consent from the participants' legal guardian/next of kin was not required to participate in this study in accordance with the national legislation and the institutional requirements.

## Author contributions

GS and KL conceived and designed the study. AD, DM, SL, JR, JN, and AT were involved in the primary data collection and aggregation. AM, UF, and CG oversaw the programmatic response to contained the epidemic and aggregated the routinely collected data. GS and LN had access to and analyzed the data. All authors participated in the development and provided a critical review of the reported research, approved the final report for the publication, and are accountable for the accuracy and integrity of the work.

## Funding

This study was funded in part by the Wellcome Trust by providing salary support to GS through the Henry Wellcome Fellowship (number 204693/Z/16/Z). The retrospective mapping exercise was supported by the CDC Senegal office and the WHO as part of the programmatic outbreak response.

## Conflict of interest

The authors declare that the research was conducted in the absence of any commercial or financial relationships that could be construed as a potential conflict of interest.

## Publisher's note

All claims expressed in this article are solely those of the authors and do not necessarily represent those of their affiliated organizations, or those of the publisher, the editors and the reviewers. Any product that may be evaluated in this article, or claim that may be made by its manufacturer, is not guaranteed or endorsed by the publisher.

## Author disclaimer

The findings and conclusions presented in this report are those of the authors and do not necessarily reflect the official position of the CDC or the World Health Organization. The use of trade names is for identification only and does not imply endorsement by the CDC, the Public Health Service, or the U.S. Department of Health and Human Services.

## Supplementary material

Supplementary Movie 1 Animation of the spatio-temporal dynamics of the malaria cases reported in Praia in 2017. Each point represents the location of geolocated cases in Praia and each frame shows the cases reported per month between January–December 2017. The color of each point represents the estimated Rt with circles reflecting cases that were locally acquired with triangles highlighting cases that were reported as imported from outside of Cabo Verde.

Supplementary Figure 1 Heatmap showing the likelihood of cases being associated according to the algorithm which is based on spatial and temporal proximity. Rt is estimated according to the sum of the probabilities that one case led to another. In the heatmap, each row and column represent an individual with the color corresponding to the probability that the infections in the individuals are likely to be connected.

Supplementary Figure 2 Histogram showing the distribution of the estimated R_T_.

Supplementary Figure 3 Classification tree and confusion matrix for land use classification of high-resolution satellite imagery.

Supplementary Figure 4 Diagnostic for the best model explaining R_T_ values in Praia.

Supplementary Figure 5 Effects of significant covariates in spatial model to predict Rt, including bare ground **(top)**, distance from water courses in meters **(middle)** and altitude **(bottom)**.

Supplementary Table 1 Comparison of geostatistical models of R_T_ in Praia, at the four different scales. AIC, Akaike's Information Criterion.

Supplementary File 1 Completed RECORD checklist outlining the reporting of routinely collected data.

## References

[1] Smith DL Battle KE Hay SI Barker CM Scott TW McKenzie FE. Ross, Macdonald, and a theory for the dynamics and control of mosquito-transmitted pathogens. PLoS Pathog. (2012) 8:e1002588. 10.1371/journal.ppat.1002588 22496640 PMC3320609

[2] Nishiura H Chowell G. The effective reproduction number as a prelude to statistical estimation of time-dependent epidemic trends. In: Castillo-Chavez C Chowell G Hayman JM Bettencourt LMA, editors. Mathematical and Statistical Estimation Approaches in Epidemiology. Dordrecht: Springer Netherlands (2009), p. 102–21. 10.1007/978-90-481-2313-1_5

[3] Enahoro I Eikenberry S Gumel AB Huijben S Paaijmans K. Long-lasting insecticidal nets and the quest for malaria eradication: a mathematical modeling approach. J Math Biol. (2020) 81:113–58. 10.1007/s00285-020-01503-z 32447420

[4] Nankabirwa JI Arinaitwe E Rek J Kilama M Kizza T Staedke SG. Malaria transmission, infection, and disease following sustained indoor residual spraying of insecticide in Tororo, Uganda. Am J Trop Med Hyg. (2020) 103:1525–33. 10.4269/ajtmh.20-0250 32700666 PMC7543828

[5] Smith DL McKenzie FE Snow RW Hay SI. Revisiting the basic reproductive number for malaria and its implications for malaria control. PLoS Biol. (2007) 5:e42. 10.1371/journal.pbio.0050042 17311470 PMC1802755

[6] Bhatt S Weiss DJ Cameron E Bisanzio D Mappin B Dalrymple U. The effect of malaria control on Plasmodium falciparum in Africa between 2000 and 2015. Nature. (2015) 526:207–11. 10.1038/nature15535 26375008 PMC4820050

[7] Namango IH Marshall C Saddler A Ross A Kaftan D Tenywa F. The Centres for Disease Control light trap (CDC-LT) and the human decoy trap (HDT) compared to the human landing catch (HLC) for measuring *Anopheles* biting in rural Tanzania. Malar J. (2022) 21:181. 10.1186/s12936-022-04192-9 35690745 PMC9188237

[8] Rodriguez-Barraquer I Arinaitwe E Jagannathan P Kamya MR Rosenthal PJ Rek J. Quantification of anti-parasite and anti-disease immunity to malaria as a function of age and exposure. eLife. (2018) V1:e35832. 10.7554/eLife.35832.045 30044224 PMC6103767

[9] van den Hoogen LL Stresman G Présumé J Romilus I Mondélus G Elismé T. Selection of antibody responses associated with *Plasmodium falciparum* infections in the context of malaria elimination. Front Immunol. (2020) 11:928. 10.3389/fimmu.2020.00928 32499783 PMC7243477

[10] Stresman G Sepúlveda N Fornace K Grignard L Mwesigwa J Achan J. Association between the proportion of *Plasmodium falciparum* and *Plasmodium vivax* infections detected by passive surveillance and the magnitude of the asymptomatic reservoir in the community: a pooled analysis of paired health facility and community data. Lancet Infect Dis. (2020) 20:953–63. 10.1016/S1473-3099(20)30059-1 32277908 PMC7391005

[11] DePina AJ Niang EHA Barbosa Andrade AJ Dia AK Moreira A Faye O. Achievement of malaria pre-elimination in Cape Verde according to the data collected from 2010 to 2016. Malar J. (2018) 17:236. 10.1186/s12936-018-2376-4 29914468 PMC6006831

[12] DePina AJ Stresman G Barros HSB Moreira AL Dia AK Furtado UD. Updates on malaria epidemiology and profile in Cabo Verde from 2010 to 2019: the goal of elimination. Malar J. (2020) 19:380. 10.1186/s12936-020-03455-7 33097051 PMC7585190

[13] van den Hoogen LL Bareng P Alves J Reyes R Macalinao M Rodrigues JM. Comparison of commercial ELISA kits to confirm the absence of transmission in malaria elimination settings. Front Public Health. (2020) 8:480. 10.3389/fpubh.2020.00480 33014975 PMC7509087

[14] Da Veiga Leal S Ward D Campino S Benavente ED Ibrahim A Claret T. Drug resistance profile and clonality of Plasmodium falciparum parasites in Cape Verde: the 2017 Malaria Outbreak. Malar J. (2021) 20:172. 10.1186/s12936-021-03708-z 33789667 PMC8011132

[15] DePina AJ Andrade AJB Dia AK Moreira AL Furtado UD Baptista H. Spatiotemporal characterisation and risk factor analysis of malaria outbreak in Cabo Verde in 2017. Trop Med Health. (2019) 47:3. 10.1186/s41182-018-0127-4 30636920 PMC6323763

[16] Routledge I Chevéz JER Cucunubá ZM Rodriguez MG Guinovart C Gustafson KB. Estimating spatiotemporally varying malaria reproduction numbers in a near elimination setting. Nat Commun. (2018) 9:2476. 10.1038/s41467-018-04577-y 29946060 PMC6018772

[17] Routledge I Unwin HJT Bhatt S. Inference of malaria reproduction numbers in three elimination settings by combining temporal data and distance metrics. Sci Rep. (2021) 11:14495. 10.1038/s41598-021-93238-0 34262054 PMC8280212

[18] Wessel B. TanDEM-X Ground Segment - DEM Products Specification Document. Oberpfaffenjofen: D. EOC (2018).

[19] Liaw A Wiener M. Classification and regression by randomForest. R News. (2002) 2:18–22.

[20] Therneau T Atkinson B. Recursive partitioning and regression trees, in *R Package*. CRAN (2019).

[21] Brooks ME. KristensenK van Benthem KJ Magnusson A Berg CW Nielsen A. glmmTMB Balances speed and flexibility among packages for zero-inflated generalized linear mixed modeling. R J. (2017) 9:378–400. 10.32614/RJ-2017-066

[22] Benchimol EI Smeeth L Guttmann A Harron K Moher D Petersen I. The reporting of studies conducted using observational routinely-collected health data (RECORD) statement. PLoS Med. (2015) 12:e1001885. 10.1371/journal.pmed.1001885 26440803 PMC4595218

[23] Bousema T Drakeley C. Epidemiology and infectivity of *Plasmodium falciparum* and *Plasmodium vivax* gametocytes in relation to malaria control and elimination. Clin Microbiol Rev. (2011) 24:377–410. 10.1128/CMR.00051-10 21482730 PMC3122489

[24] Reiner RC Le Menach A Kunene S Ntshalintshali N Hsiang MS Perkins TA. Mapping residual transmission for malaria elimination. Elife. (2015) 4:e09520. 10.7554/eLife.09520 26714110 PMC4744184

[25] Service MW. Mosquito (Diptera: Culicidae) dispersal–the long and short of it. J Med Entomol. (1997) 34:579–88. 10.1093/jmedent/34.6.579 9439109

[26] Huestis DL Dao A Diallo M Sanogo ZL Samake D Yaro AS. Windborne long-distance migration of malaria mosquitoes in the Sahel. Nature. (2019) 574:404–8. 10.1038/s41586-019-1622-4 31578527 PMC11095661

[27] Andolina C Rek JC Briggs J Okoth J Musiime A Ramjith J. Sources of persistent malaria transmission in a setting with effective malaria control in eastern Uganda: a longitudinal, observational cohort study. Lancet Infect Dis. (2021) 21:1568–78. 10.1016/S1473-3099(21)00072-4 34146476 PMC8554388

[28] Stresman G Whittaker C Slater HC Bousema T Cook J. Quantifying *Plasmodium falciparum* infections clustering within households to inform household-based intervention strategies for malaria control programs: an observational study and meta-analysis from 41 malaria-endemic countries. PLoS Med. (2020) 17:e1003370. 10.1371/journal.pmed.1003370 33119589 PMC7595326

[29] Nasir SMI Amarasekara S Wickremasinghe R Fernando D Udagama P. Prevention of re-establishment of malaria: historical perspective and future prospects. Malar J. (2020) 19:452. 10.1186/s12936-020-03527-8 33287809 PMC7720033

[30] Smith DL Hay SI Noor AM Snow RW. Predicting changing malaria risk after expanded insecticide-treated net coverage in Africa. Trends Parasitol. (2009) 25:511–6. 10.1016/j.pt.2009.08.002 19744887 PMC2768685

[31] Dharmawardena P Premaratne R Wickremasinghe R Mendis K Fernando D. Epidemiological profile of imported malaria cases in the prevention of reestablishment phase in Sri Lanka. Pathog Glob Health. (2022) 116:38–46. 10.1080/20477724.2021.1951556 34263705 PMC8812787

[32] Churcher TS Cohen JM Novotny J Ntshalintshali N Kunene S Cauchemez S. Public health. Measuring the path toward malaria elimination. Science. (2014) 344:1230–2. 10.1126/science.1251449 24926005 PMC4340075

[33] Woolhouse MEJ Dye C Etard JF Smith T Charlwood JD Garnett GP. Heterogeneities in the transmission of infectious agents: implications for the design of control programs. Proc Natl Acad Sci U S A. (1997) 94:338–42. 10.1073/pnas.94.1.338 8990210 PMC19338

[34] Smith DL Perkins TA Reiner RC Jr Barker CM Niu T Chaves LF. Recasting the theory of mosquito-borne pathogen transmission dynamics and control. Trans R Soc Trop Med Hyg. (2014) 108:185–97. 10.1093/trstmh/tru026 24591453 PMC3952634

