# Supplementary material for: Factors related to human-vector contact that modify the likelihood of malaria transmission during a contained Plasmodium falciparum outbreak in Praia, Cabo Verde
Source: Front Epidemiol. 2022 Nov 25;2:1031230. doi: 10.3389/fepid.2022.1031230 (PMC10910924; doi:10.3389/fepid.2022.1031230)

**Supplemental Information**

**Suppl Table 1** – Comparison of geostatistical models of R_T_ in Praia, at the four different scales. AIC: Akaike’s Information Criterion.

| **Model** | **AIC** | **ΔAIC** |
| --- | --- | --- |
| 25m | 768.15 | 0 |
| 50m | 771.20 | 3.05 |
| 75m | 770.33 | 2.18 |
| 100m | 772.36 | 4.21 |

**Suppl Figure 1** – Heatmap showing the likelihood of cases being associated according to the

algorithm which is based on spatial and temporal proximity. Rt is estimated according to the sum of the probabilities that one case led to another. In the heatmap, each row and column represent an individual with the color corresponding to the probability that the infections in the individuals are likely to be connected.

**Suppl Figure 2** - Histogram showing the distribution of the estimated R_T_.

**
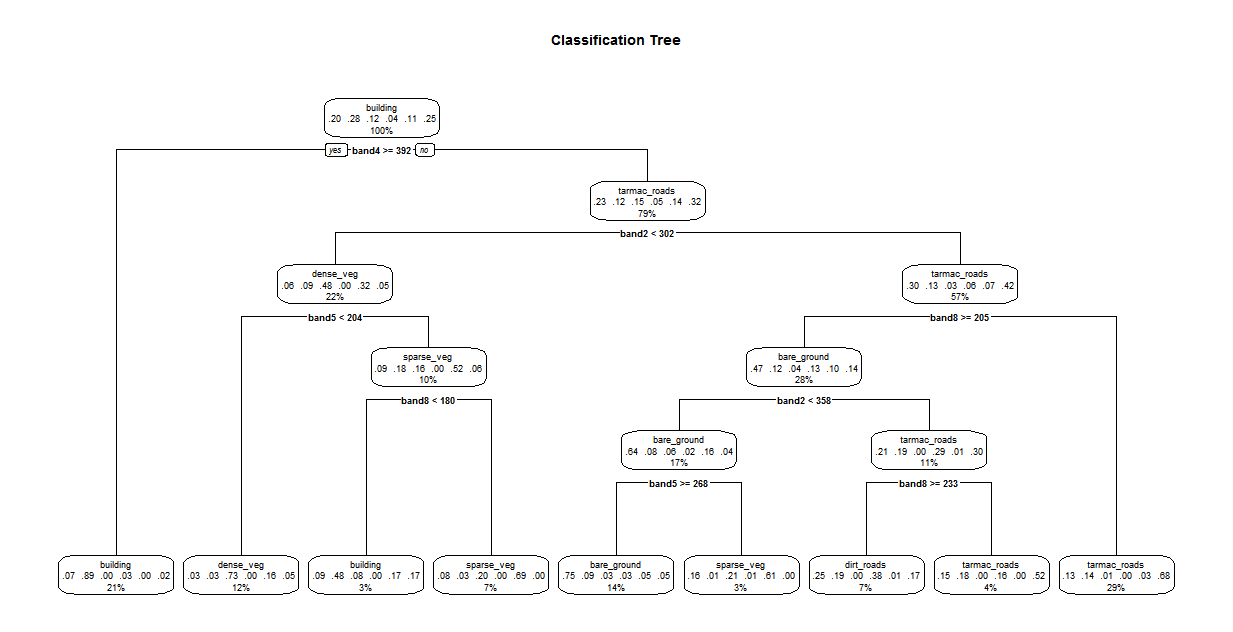
Suppl Figure 3** – Classification tree and confusion matrix for land use classification of high-resolution satellite imagery.


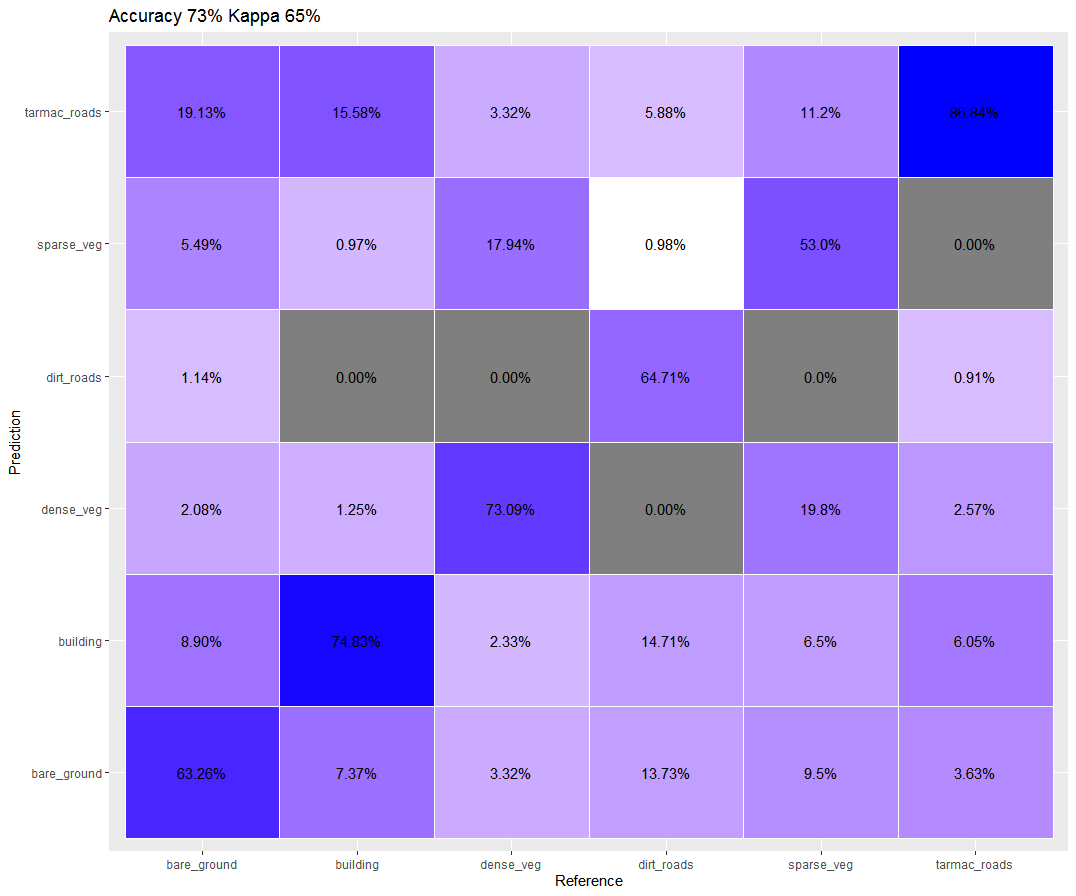


**Suppl Figure 4 –**Diagnostic for the best model explaining R_T_ values in Praia

**
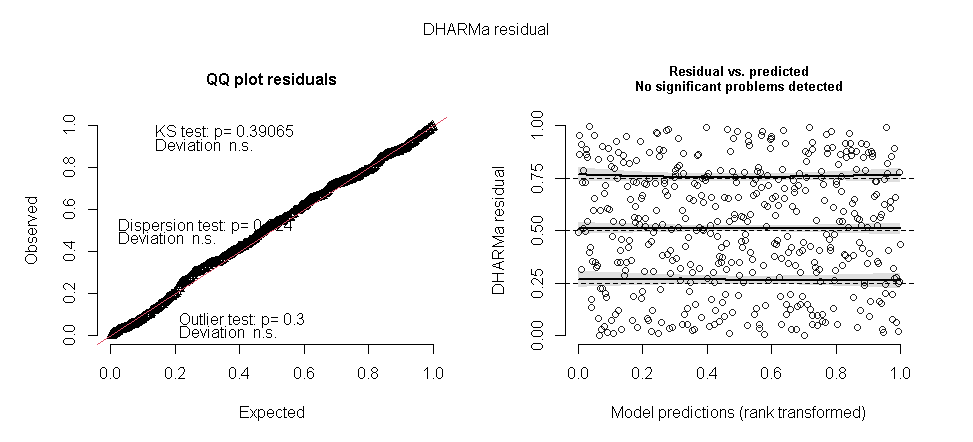
**

**Suppl Figure 5** – Effects of significant covariates in spatial model to predict Rt, including bare ground (top), distance from water courses in meters (middle) and altitude (bottom).


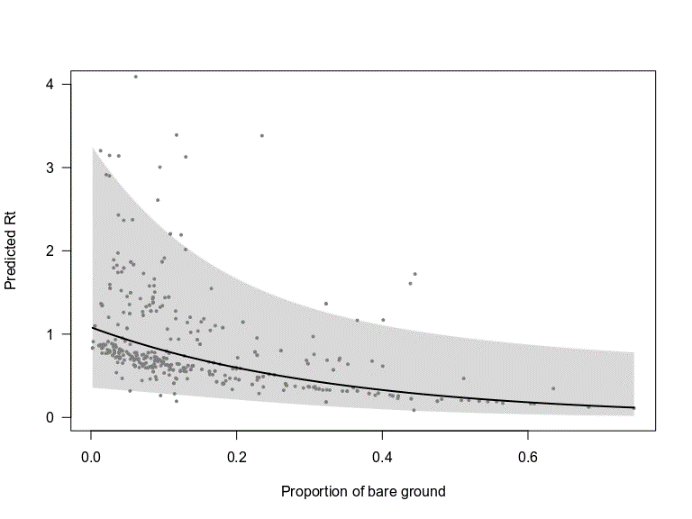


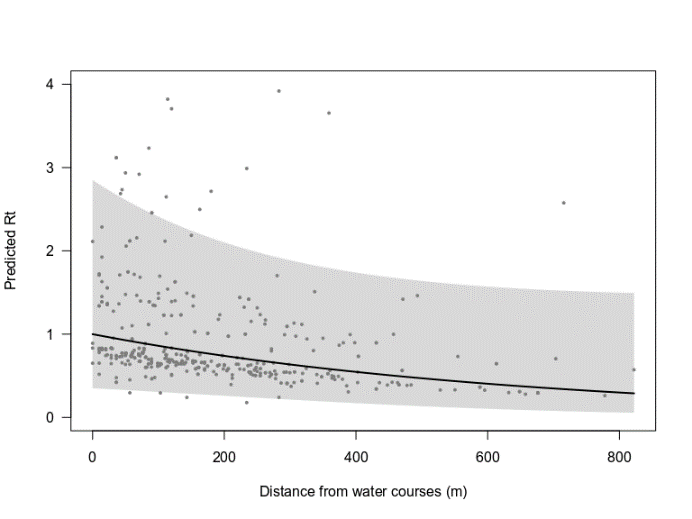


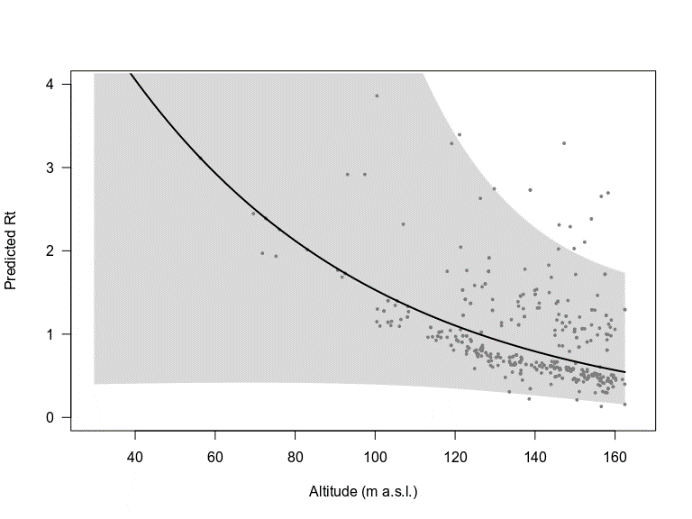

Supplement: Supplementary Figure 1 — Heatmap showing the likelihood of cases being associated according to the algorithm which is based on spatial and temporal proximity. Rt is estimated according to the sum of the probabilities that one case led to another. In the heatmap, each row and column represent an individual with the color corresponding to the probability that the infections in the individuals are likely to be connected. [file Data_Sheet_1.docx]
